# Effects of post-saccadic oscillations on visual processing times

**DOI:** 10.1371/journal.pone.0302459

**Published:** 2024-05-29

**Authors:** Emsal Llapashtica, Tong Sun, Kenneth T. V. Grattan, John L. Barbur

**Affiliations:** 1 The Henry Wellcome Laboratories for Vision Science, Centre for Applied Vision Research, School of Health & Psychological Sciences, University of London, London, United Kingdom; 2 Department of Engineering, School of Science and Technology, University of London, London, United Kingdom; The Ohio State University, UNITED STATES

## Abstract

Saccadic eye movements enable us to search for the target of interest in a crowded scene or, in the case of goal-directed saccades, to simply bring the image of the peripheral target to the very centre of the fovea. This mechanism extends the use of the superior image processing performance of the fovea over a large visual field. We know that visual information is processed quickly at the end of each saccade but estimates of the times involved remain controversial. This study aims to investigate the processing of visual information during post fixation oscillations of the eyeball. A new psychophysical test measures the combined eye movement response latencies, including fixation duration and visual processing times. When the test is used in conjunction with an eye tracker, each component that makes up the ‘integrated saccade latency’ time, from the onset of the peripheral stimulus to the correct interpretation of the information carried by the stimulus, can be measured and the discrete components delineated. The results show that the time required to process and encode the stimulus attribute of interest at the end of a saccade is longer than the time needed to carry out the same task in the absence of an eye movement. We propose two principal hypotheses, each of which can account for this finding. 1. The known inhibition of afferent retinal signals during fast eye movements extends beyond the end point of the saccade. 2. The extended visual processing times measured when saccades are involved are caused by the transient loss of spatial resolution due to eyeball instability during post-saccadic oscillations. The latter can best be described as retinal image smear with greater loss of spatial resolution expected for stimuli of low luminance contrast.

## 1. Introduction

Since only the very central region of the retina (the fovea centralis), which subtends just under 2° of visual angle [1] and is centred on the point of regard has superior performance in functions of visual acuity, contrast sensitivity, motion and flicker detection and colour discrimination, the saccadic eye movement system is essential in order to achieve high performance over a large visual field. Humans can generate as many as three saccades every second, searching the surrounding environment and acquiring new information. Each saccadic eye movement–as the eye undergoes rapid acceleration–causes fast retinal image movements. During these rapid movements our vision is largely suppressed. Complex oculomotor movements and stages of visual processing are involved, but we normally take all these for granted since the processes involved are largely pre-attentive and effortless. Saccadic suppression can affect the perception of visual stimuli presented during the movement, and may also outlast the end of the saccade [2,3]. Sustained lens and eyeball oscillatory movements produce retinal image smear that can cause post-saccadic loss of visual acuity [4,5].

It is only during the last decade with proliferation of video based eye trackers that post saccadic oscillatory movements (PSO) have become of greater interest in vision research [6–10], despite the fact that the perceptual consequences of lens oscillation have been described a quarter of a century ago [4]. Studies that employed search coil techniques to measure eye movements have also provided some evidence to suggest that the eyeball continues to move at the end of a saccade [11–13]. It is widely acknowledged that saccades are associated with overshoots and ‘ringing’ because the sudden initial torque to initiate the movement is always large and the forces involved in bringing the eyeball to rest cause damped oscillations. The large, initial torque applied to the eyeball is needed to overcome the viscosities of the orbital tissues [12,14–16]. A large torque generates high initial acceleration and large rotational speeds which are needed to overcome the inertia of the eyeball and to minimise the time spent in flight. Position accuracy at the end of the saccade is often affected by the rotational speeds involved and the properties of the stimulus [17]. When the subject can only make use of retinal projections to midbrain nuclei, the saccadic end point tends to be determined by the ‘centre of gravity’ of the light flux distribution in the stimulus [18]. Irrespective of the accuracy of saccade end point, position uncertainty is always involved during the damped oscillatory motion which follows the rapid deceleration phase at the end of each saccade which in turn can affect visual processing times. Successive studies have confirmed that overshoots have neural origin [14,19,20], but more importantly, the evidence also reveals a close relationship between the eye velocity and the overshoot amplitude suggesting that post movements can be predicted from the measured velocity profiles [13,19]. Other studies have shown that overshoots can retain large velocities, often as large as 100°/sec. These are greater than vergence drift velocities which are often too small (≤ 20°/sec) to compromise visual acuity significantly [11,21], or to generate perceived motion, largely as a result of spatiotemporal properties of motion detection mechanisms [22]. Surprisingly, none of these studies mention specifically whether saccade overshoots cause transient impairments in the ability to detect, resolve and discriminate visual stimuli, or the potential effects these may have on visual processing times. It has, however, been shown that when high speeds (> 100°/sec), eyeball rotation is involved, small, low contrast visual stimuli become more difficult to see [23]. While this result alone provides a clear indication that PSOs can cause transient impairment in our ability to process visual information at the end of each saccade, only a few of the studies mentioned above, have addressed the potential effects of eyeball instabilities. This is of particular interest especially, when considering that when exploring the environment, eyeball fixations account for ~ 80% of the total visual search time [24]. In this study we asked if image smear caused by eyeball instability at the end of a saccade affects visual processing times. To test this hypothesis, we carried out a set of experiments designed to estimate the direct time needed to process the same visual attribute with or in the absence of saccadic eye movements. We used the EMAIL (Eye Movements And Integrated Latencies) test described here in combination with the EyeLink 1000 video-based, eye tracker system, to demonstrate that the fixation durations needed to process specific visual information at the end of a saccade are longer than what the visual system needs for the same visual task, when no eye movements are involved. We also test whether saccadic suppression or image smear contributes most to the increased visual processing times by investigating how the luminance contrast of the stimulus affects visual processing times when saccades are involved. The EMAIL test measures the time the subject needs to detect the onset of the peripheral stimulus, to generate the eye movement needed and to process the stimulus attribute of interest at the end of the saccade. The addition of eye tracking enabled us to separate each component that makes up the total ‘Integrated Saccade Latency’ (ISL) and to demonstrate that the increase in visual processing times when saccades are involved matches well the durations of PSOs.

## 2. Methods

**Fig 1:**
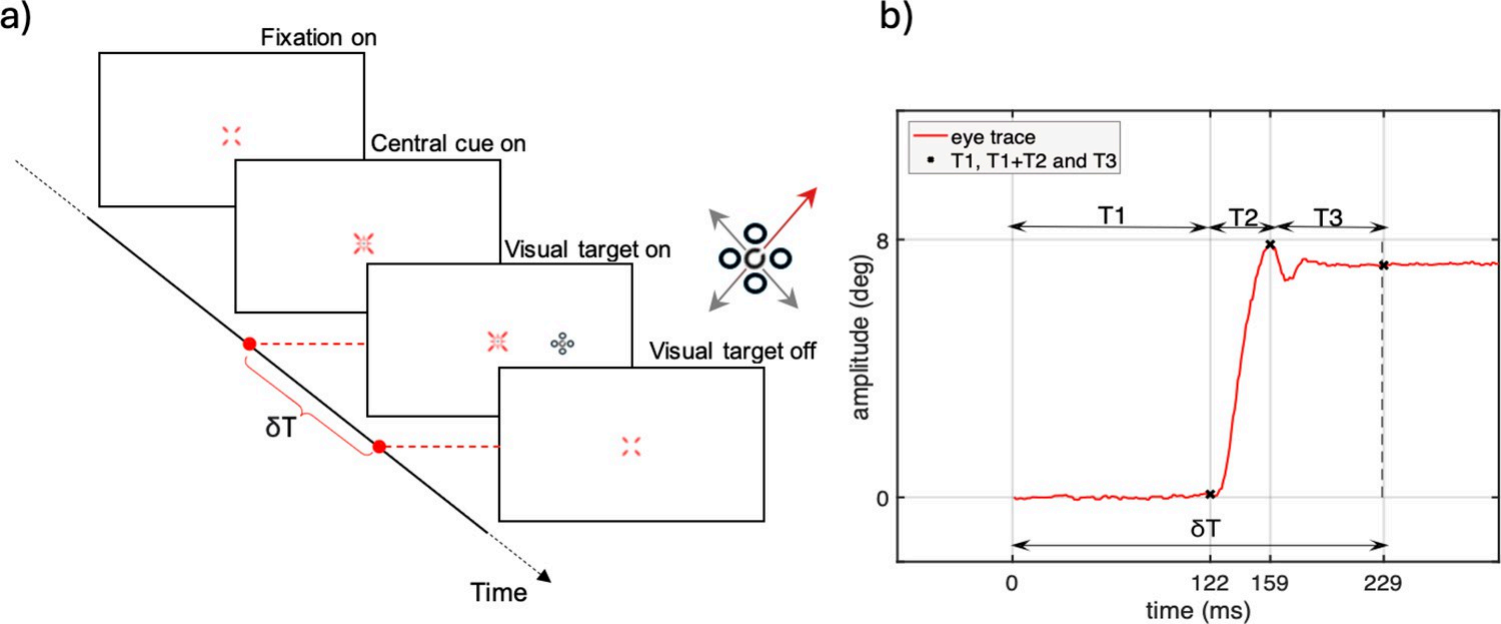
Schematic representation of the timeline employed in the EMAIL test. (a). The onset of the guides presented in the centre of the screen attract the subject’s fixation and signal the start of the experiment. Shortly afterwards a cue appears at the centre of the guides, indicating the need for steady fixation. In the first experiment, the test stimulus is presented in the periphery along the horizontal meridian, randomly on either side of fixation, for a fixed time, T, selected by the staircase. The subject’s task is to saccade towards the target and to register the orientation of the gap in the central ring (see a). The subject is then required to press one of four response buttons to indicate the position of the gap, or to simply guess when unable to decide. The time the subject takes to press the appropriate button affects only the decision response time and not the Integrated Saccade Latency time (δT). A four-alternative, forced-choice (4AFC) staircase procedure with variable step sizes is used to measure, δT. The latter represents the time the subject needs to achieve 71% probability of a correct response. The staircase employed varies the stimulus presentation time, T, using a ‘2-down, 1-up’ procedure. δT is then calculated by averaging the last 12 staircase reversals. No eye-tracker is needed to measure, δT. The addition of an eye-tracker does, however, make it possible to separate the various components that make up the ISL time. A typical record of a single rightward saccade, as recorded with the EyeLink1000 is shown in (b). The signal depicts all three saccade parameters where the latency is denoted as T1 and corresponds to the time required to detect the stimulus and prepare the saccadic eye movement. The saccade duration is denoted by T2. T3 represents the remaining stimulus time the subject can use to process the information of interest in the visual stimulus. (b) also shows the components T1, T2 and T3 which make up δT. Additional experiments were also carried out with the stimulus presented at the point of fixation in the absence of saccadic eye movements. The subject’s task remained unchanged, but no eye movements were involved.

The EMAIL test developed for this study has not been described previously. The test was designed to measure the time needed to carry out an appropriate saccadic eye movement to a stimulus presented in the periphery of the visual field. The stimulus can have one of a number of different visual attributes which the subject must process at the end of the saccade in order to generate the correct response. The EMAIL test runs on the AVOT system (City Occupational Ltd, Cumbria UK). The AVOT equipment employs a 10 bit NEC MultiSync display (Model NEC PA241W) with a typical response time of 8 ms. The luminance and the chromaticity of each primary color were calibrated using a Konica CS-2000A telespectroradiometer (Konica Minolta Inc.) over a spectral range of 380 to 780 nm. The EMAIL program runs on a Dell Optiplex 3080 under Windows 10 operating system. The test does not require eye-tracking equipment, is inexpensive, requires no calibration and also measures the visual processing time that relates specifically to the stimulus attribute of interest. The subject’s decision Response Time (RT), which includes a spatial coordination task, and the speed of the motor response is also measured. The latter parameter is of interest in some occupations. Conventionally, under controlled conditions, the saccadic responses are measured by controlling accurately the timing and location of the target that initiates the saccade; the subject’s performance is determined from eye movement recordings. Based on a similar principle, the EMAIL test also initiates the saccades, but the subject’s performance is determined by measuring a single variable, δT. The latter represents the shortest time the subject needs to detect the peripheral target, generate an appropriate eye movement and process the specific stimulus attribute of interest at the end of the saccade. A four-alternative, forced-choice (4AFC) staircase procedure with variable step sizes is used to measure, δT. The staircase employed varies the stimulus presentation time, T, using a ‘2-down, 1-up’ procedure and yields the time the subject needs to achieve 71% correct response rate [25]. For simplicity, the threshold time (δT) estimated in this way is described as the Integrated Saccade Latency (ISL). The test employs an overlap paradigm to trigger visually guided saccades. This paradigm is desirable because the temporal relationship between the subject’s point of regard and the test target resembles the usual occurrence of novel visual stimuli that often become detectable in the visual periphery under natural viewing conditions. Fig 1 illustrates the experimental timeline employed in the test. Each trial begins with a brief, intermittent appearance of the fixation target at the centre of diagonal guides. The guides help delineate the centre of the screen, i.e., the expected location of the fixation stimulus. The sudden appearance of a conspicuous, centre fixation target attracts pre-attentively the subject’s point of regard. Following a brief time, ranging from 400 to 800ms, the central cue appears in the middle of the cross, to indicate to the subject the need to maintain central fixation. This is then followed by the onset of a peripheral target at one of two, randomly chosen, predetermined locations, either to the right or to the left of fixation, along the horizontal meridian in the visual field. The test target consists of a Landolt C with a gap size of four arc minutes surrounded by four ring distractors of similar size. During each stimulus presentation, the position of the gap in the Landolt C at the centre of the group is selected randomly to correspond to one of the four diagonal directions, as shown in Fig 1A. The subject is required to saccade to the stimulus, and to ‘register’ the orientation of the gap in the Landolt ring (Fig 1A). The staircase controls the time, T, the stimulus is presented on the screen. At the end of this time, the screen returns to a uniform background and the subject’s final task is to press one of four buttons on a numeric keypad to indicate the location of the gap. The four keypad buttons employed in the EMAIL test are raised above all other keys and selected to form a square with its corners matching the orientation of the four gap locations in the stimulus. The subject’s response time is also recorded and provides a measure of the time the subject needs to link mentally the position of the gap with the corresponding button location and to implement the motor response. The target configuration employed was designed to cause large ‘visual crowding’ to ensure that the subject is completely unable to carry out the visual task in the periphery in the absence of an eye movement [26–29]. To carry out the task successfully, an appropriate saccade is needed to bring the point of regard onto the target and the stimulus presentation time, T, must be long enough for the subject to process the visual information at the end of the saccade. An important advantage of this measurement technique is that the estimated ISL includes the time needed to process and encode the information of interest in the foveal stimulus at the end of each saccade. By using the test in conjunction with the eye tracker, it becomes possible to separate each component that makes up the ISL (Fig 1B). Using this approach, the stimulus durations needed to process the stimulus at the end of each saccade can be measured accurately. The stimulus attribute of interest was the location of the gap in the Landolt ring. Other stimulus attributes such as the location of a coloured stimulus buried in dynamic luminance contrast noise which isolates the use of colour signals, a rapidly flickering quadrant in a square stimulus or the diagonal direction of motion of a moving stimulus defined by either luminance or colour contrast can also be employed to measure differences in cortical processing times for each of the stimulus attributes listed above [30–33].

### 2.1. Subjects

The findings presented here are based on experiments carried out in the Centre for Applied Vision Research at City, University of London. The method and testing procedures were approved by the Research and Ethical Committee of the University. All participants provided written informed consent and were financially compensated for their time.

Six subjects (three men and three women) ranging from 22 to 50 years took part in the study between January 2020 to December 2022. All participants had normal or corrected-to-normal visual acuity. The study was conducted according to the principles defined in the declaration of Helsinki.

### 2.2. Experimental procedure

Two related experiments were carried out with each participant. The first experiment was designed to measure ISL values, while the second experiment combined the EMAIL test with the eye-tracker and measured the probability of a correct response for a number of discrete stimulus presentation times, selected to fall both above and below the subject’s ISL time determined with the EMAIL test.

The experiments were carried out in a darkened room. The subject viewed the visual display from a distance of 80 cm, and a chin rest was employed to stabilise the subject’s head position. The uniform background field had a luminance of 32 cd/m^2^ and the luminance contrast of the Landolt ring stimulus and the distracter rings was set to: (1) 75% and (2) 15%. Each subject completed three repeat threshold measurements for each condition in a single session. All trials ended on completion of 17 ‘reversals’ within the staircase procedure which involved approximately 50 to 65 stimulus presentations per trial. Each block of 3 trials took approximately 20 min to complete. We ran the following three experimental sequences to measure the stimulus durations needed to process the same visual attribute with or in the absence of saccadic eye movements.

1. Sequence 1. The subject’s ISL values were measured for a stimulus of either 15% or 75% luminance contrast, presented randomly in the periphery on either side of fixation at an eccentricity of 8°.
2. Sequence 2. The subject had to carry out essentially the same test with fixed stimulus presentation times selected to fall within 20ms, both above and below the subject’s measured ISL time. The corresponding eye movements for each discrete stimulus presentation time were recorded in every presentation. This procedure made it possible to determine the probability of a correct response for each discrete stimulus presentation time.
3. Sequence 3. The second experiment was replicated to measure the stimulus presentation time the subject needs to achieve a correct response in the absence of eye-movements with a stimulus of 15% luminance contrast presented at the point of regard (i.e. at 0° eccentricity). This was done in order to estimate the visual processing time in the absence of eye movements.

Although the experimental method developed for this study has many advantages, the hardware employed in the EMAIL test limits the shortest stimulus time that could be achieved to ~25ms. In order to discover how this limitation arises, we measured the actual stimulus time by placing a light-sensitive, photodiode detector directly on the stimulus display. A simple circuit was built to measure and record the actual stimulus time on the display for a given set time. A series of measurements were made for different stimulus set times (as specified in the program).

**Fig 2:**
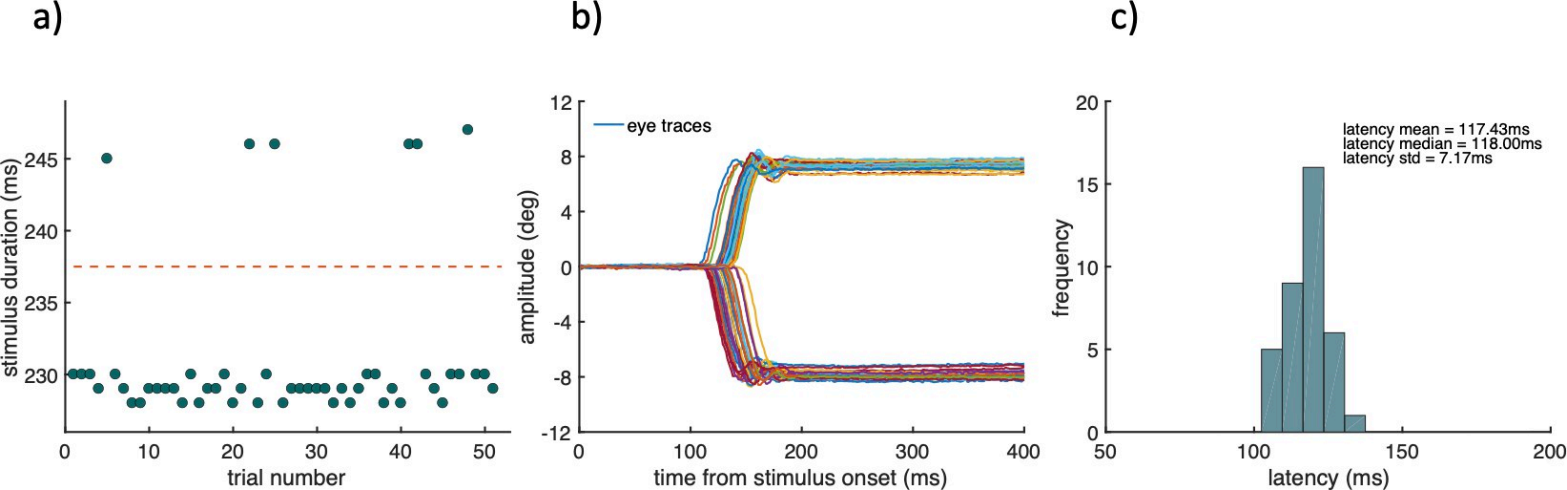
Stimulus duration times as measured on the display (a), eye trace recordings (b) and the corresponding response latency histogram (c). (a) Stimulus durations as recorded with the photodiode system on the 60Hz visual display for a constant stimulus presentation time of 230ms. As the stimulus is presented on the screen, the photodiode and the associated electronics generate a TTL signal that begins on stimulus onset and terminates on stimulus offset. This arrangement enables the measurement of the actual time of the stimulus on the screen. Leftward and rightward eye traces are shown in (b). The start of each saccade (shown by the coloured lines) is synchronised with the onset of the stimulus. The frequency histogram of saccadic latencies is shown in (c) with the corresponding mean, median and standard deviation.

The results revealed three findings of interest. First, the shortest stimulus time that can be achieved using the EMAIL program corresponded to two display frames. Second, with a frame rate of 60 Hz, each frame was only held active for just over 8ms within the 16.66ms window. This observation accounts for the shortest stimulus time of ~ 25ms (which corresponds to two frames). Third, the lag time was approximately constant so that each set time corresponded to a fixed number of frames. A small percentage of stimulus presentations needed to estimate the ISL time (often less than 5%) differed by one frame (see Fig 2A). This limitation has only a small effect of the ISL values measured with the EMAIL test and no effect on the ISL values estimated from the combined EMAIL / eye-tracker tests which made use of the actual measured stimulus times on the display and not the set times specified in the program. When longer stimulus presentation times are employed, the display timing errors are of less concern. The shortest time of 25ms that could be achieved was a limitation since it has been shown that when high contrast stimuli are employed in complex scenes, the visual system can process and extract reliably the information in the stimulus, even for presentation times shorter than 20ms [34]. The time needed to gain enough information to process the visual attribute of interest in our experiments increased well above 25ms for the lowest contrast stimulus. We were therefore able to measure reliably the ISL times in central vision when the stimulus contrast was 15%. Comparison of foveal and peripheral visual processing times when measured with stimuli of 15% contrast made it possible to establish how visual processing times change when saccades are involved.

### 2.3. Eye movement recordings

Eye movements in the second experiment were measured using the EyeLink 1000 eye-tracker at a sampling frequency of 1000 Hz. The absolute spatial resolution of the system following initial calibration is claimed to be ~ 0.5°, with a much higher relative spatial resolution of ~ 0.1°. The experiments were performed binocularly, but the eye movement traces were measured only in the right eye. Movement of the subject’s head was minimised using chin and forehead rests with the eye located 80cm in front of the stimulus display. Each trial began with an EyeLink, 9-point calibration routine and was followed by a validation check to evaluate the gaze accuracy. Following satisfactory calibration, the testing session began. Each test involved a minimum of 50 stimulus presentations in order to estimate reliably the time course of the saccade and the probability of a correct response. Three successive test runs were needed to complete each of the second and third set of experiments. For the first trial, the stimulus duration corresponded to the subjects’ ISL time measured with the EMAIL test, whereas during the second and third runs, stimulus durations were both above and below the measured ISL time. A Weibull function was then fitted to the measured data in order to estimate the time the subject requires to achieve the desired probability of 71% correct response which matches the expected probability for the time, δT, measured with the EMAIL test. For the combined EMAIL / Eye-tracker experiments we also employed a custom-made photodiode attached to a corner of the display to capture the exact stimulus duration time. This arrangement made it possible to measure accurately the actual stimulus time on the display. The eye movement recordings were synchronised with the onset and offset times of the stimulus as measured with the photodiode.

### 2.4. Data analysis

A custom-made algorithm was used to extract the points of interest from each eye trace in the second set of experiments based on the combined EMAIL and eye tracker tests, as illustrated in Fig 1B. All trials with blinks were removed automatically from the analysis. Also, saccades with latencies shorter than 60ms were classified as anticipatory and also excluded [35]. As the ISL values are subject specific, the data were analysed separately for each subject. The correct responses were adjusted for chance probability prior to fitting the data with a Weibull function. The chance probability of a correct response in the second experiment was 25%. The stimulus presentation time needed to achieve 71% correct response was then calculated for each subject from the corresponding probability of correct response curve derived using the combined EMAIL and eye-tracking experiments. This was then labelled, ISL’, to distinguish it from the integrated saccade latency (ISL) measured with the EMAIL test.

The parameters that make up the ISL´ (such as T1, T2 and T3, see Fig 1B) were estimated from eye movement recordings. The first zero crossing after the peak velocity occurred determined the end of the saccade (i.e., its amplitude) and the difference between saccade offset and onset times defined the duration of the eye-movement (T2). The saccadic latency (T1) was calculated from the onset of the target to the initiation of the saccade, and T3 was estimated by subtracting T1+T2 from ISL´. An example of stimulus duration times as measured on the display, eye trace recordings and the corresponding response latency histogram taken from one trial consisting of 40 correct responses out of 54 target presentations is shown in Fig 2. Each dot in Fig 2A represents the exact time duration of the stimulus on the visual display recorded with the photodiode. Fig 2B shows the leftward and rightward eye traces that correspond to correct responses. Fig 2C shows the statistical distribution of saccadic latencies.

**Fig 3:**
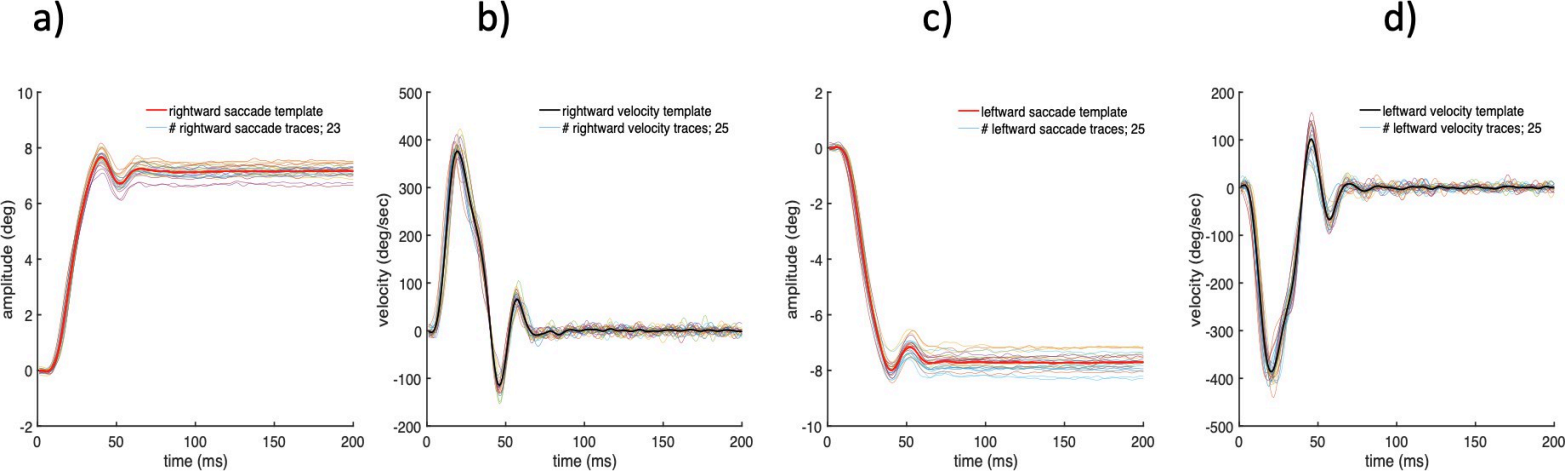
Mean saccade and velocity templates for rightward (a,b) and leftward (c,d) saccadic movements. Each thin coloured line corresponds to an individual saccade with velocity traces aligned with respect to the saccade onset time. The solid black and red lines represent the corresponding mean templates.

The subjects’ eye velocity data were also used to determine the start and end of post saccadic oscillations (PSO). The velocity data were included in the PSO analysis for two reasons. First, the change in eye velocity determines the start as well as the end of a saccade. Second, as noted in the introduction, it is the eye velocity that characterises the post saccadic movements, as it can distinguish the post saccadic drift from the faster, oscillatory movement. In Fig 3 we show ‘aligned’ saccadic and velocity trajectories that are representative of the trial above, where each saccadic trace began 1ms before saccade onset time. Note that, this alignment procedure is very useful since it eliminates the variability associated with saccade onset times (Fig 2C) and this makes possible the computation of mean templates for both saccadic and eye velocity traces. Fig 3 demonstrates clearly that each saccadic trace is indeed accompanied by post oscillatory movement as outlined by their corresponding mean saccadic and velocity templates (indicated in solid red and black lines, respectively).

**Fig 4:**
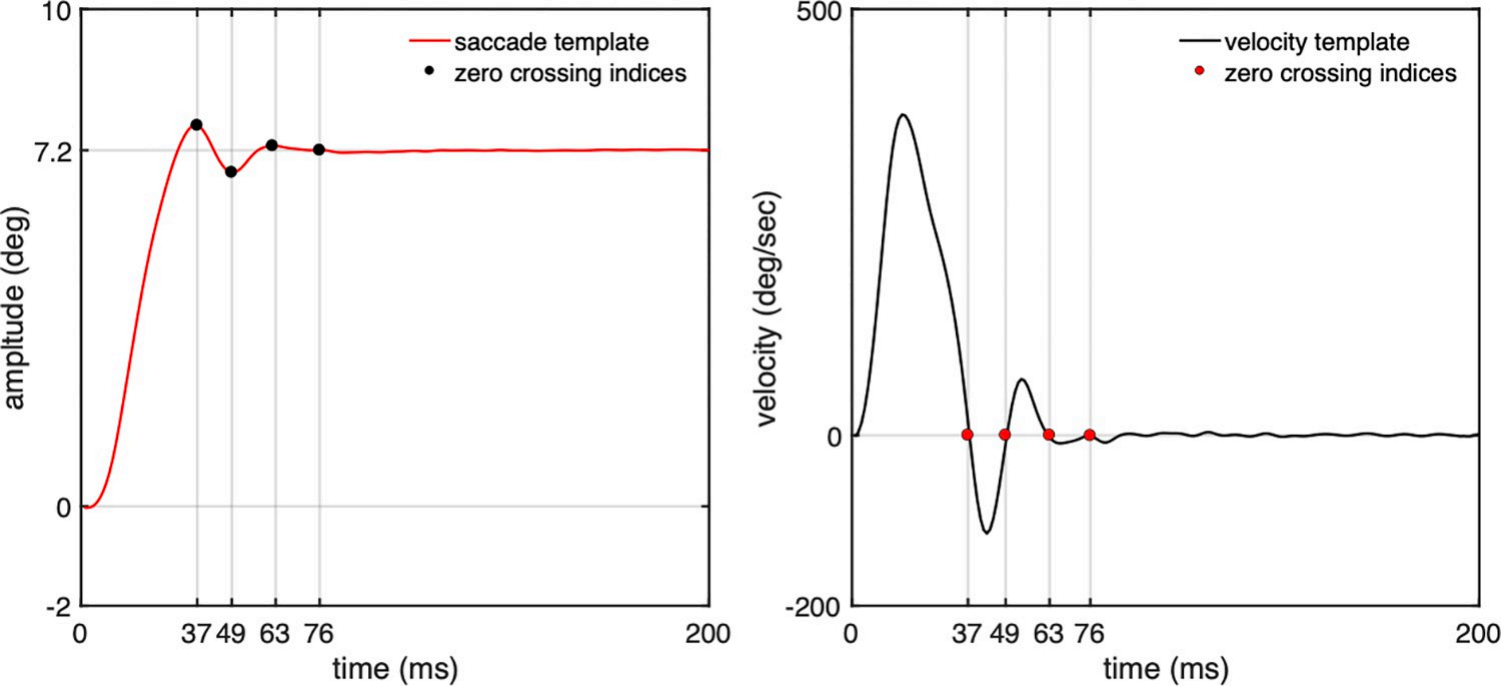
Mean saccade and velocity templates. The occurrences of max and min peaks coincide precisely with zero crossings indicated by red dots. During the movement there is a phase difference of 90° between the eye position and velocity as the latter crosses the y-axis. The difference becomes zero when the eye approaches its mean position.

Fig 3 shows both leftward and rightward mean templates to explain the tight relationship between the return velocity (i.e., first zero crossing from saccade onset) and the PSO behaviour. For clarity of presentation, Fig 4 shows only the data templates which correspond to the rightward saccades. As can be seen from Fig 4, when the first zero crossing occurs, there is an ongoing movement, but in the opposite direction (i.e., the start of the return velocity). Since the slope at any point in the saccade trajectory determines the velocity of the eyeball at that point, any change in the direction of movement is reflected clearly in both figures. Therefore, where max or min peaks (indicated as black dots) occur in saccade trajectory, the gradient, f ’(x) equals zero, thus the velocity at these points crosses the zero line (as indicated by the red dots). Also, both figures illustrate clearly that there is a 90° phase difference between the eye position and velocity—when the eye position is at maximum, the eye velocity passes through zero and vice versa. These features were present in every trace, but single traces are noisier and more difficult to analyse. Use has therefore been made of zero crossing points in each subject’s return velocity templates to determine accurately the time durations when the fast, oscillatory movement resulting from saccade overshoot ended and the eye reached its mean position.

## 3. Results

**Fig 5:**
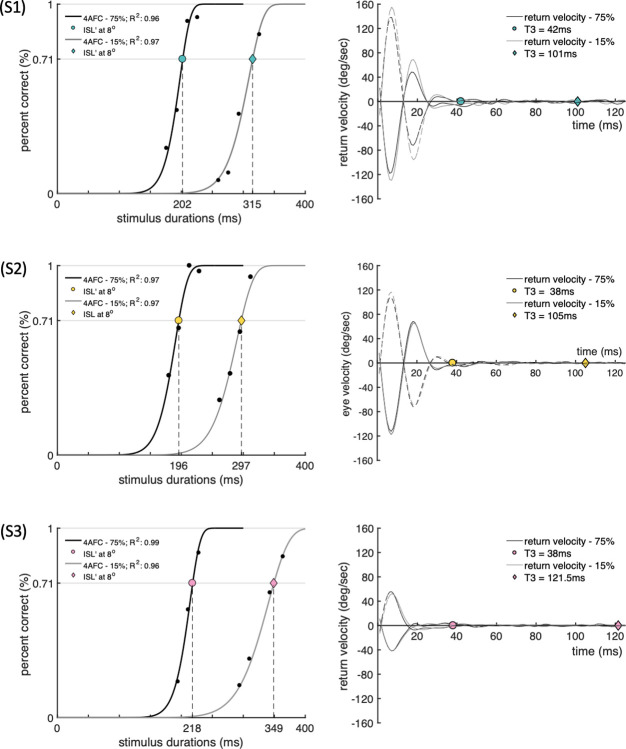
Examples of psychometric functions (left) corrected for chance probability and the corresponding return velocities (right) measured with 75% and 15% contrast stimuli for three subjects. Data are presented for three subjects. Black and grey traces are used to indicate results measured for the 75% and 15% contrast stimuli, respectively. The corresponding return velocities for leftward (dashed lines) and rightward saccades (solid lines) are shown on the right. The stimulus presentation time, ISL’, each subject needs to achieve 71% correct response is indicated by a coloured circle (75%) or a diamond (15%) with respect to stimulus onset in the left panel and with respect to the end of the saccade, in the right panel. The T3 durations are also indicated by coloured circles or diamond symbols shown on the x-axis of each figure in the right panel. Each velocity template is representative of the subject’s PSO movements that follow the end of the saccade.

The measured ISL’ thresholds and the saccade parameters that make up the ISL’ for a stimulus of 75% contrast presented peripherally at an eccentricity of 8° and for stimuli of 15% contrast, presented at the point of regard are listed in Tables 1 and 2. In Fig 5, we show superimposed psychometric functions (left panel) for each of three subjects and the corresponding return velocity templates (right panel); the black curves indicate the measurements made with 75% contrast stimulus, The results obtained with the 15% contrast stimulus are distinguished by grey lines. As expected, higher contrast stimuli evoke faster responses, with a leftward shift of the corresponding psychometric function (i.e., towards shorter stimulus durations) when compared to the equivalent psychometric curve measured with 15% contrast. On the other hand, we found that each subject’s corresponding return velocities did not vary with stimulus contrast (Fig 5, right panel). Our results demonstrate that stimulus contrast does not affect PSOs, and provided the saccade is generated following the presentation of the stimulus, the main sequence relationship remains unchanged [36]. Therefore, saccades of the same amplitude produce an invariant waveform of return velocities. In addition, this observation confirms that PSOs depend on the deceleration phase of saccades, consistent with previous findings concerning saccade overshoots [13,19]. The response latencies and visual processing times for the same saccade amplitudes do, however, depend strongly on stimulus contrast. The results reveal subject specific ISL’ thresholds which exhibit a strong dependence on stimulus contrast. The measured values ranged between 187-218ms (75%) and 287-376ms (15%). Similarly, the T1 durations also showed some subject dependency. T1 values ranged between 107-138ms (75%) and 148-185ms (15%). The higher stimulus contrast (75%) also produces shorter T3 times ranging between 36.5–46.5ms, when compared to 93-166ms (for 15% contrast). T2 durations, on the other hand, displayed the well-known main sequence characteristics and were found to be less variable and similar for both stimulus contrast levels, ranging from 38 to 43ms. The large, within and inter-subject variability in saccadic latencies is well known, but the post saccadic visual processing times have not been examined in detail in past studies and remain poorly understood. The design of the EMAIL test ensures that at the end of each saccade, the stimulus remains under foveal examination for as long as the subject needs to process reliably the stimulus feature of interest, which in our experiments is the gap in the Landolt ring and its location.

**Table 1:** All times are shown in ms and amplitudes are in degrees of visual angle.

*Contrast* *75%*	*Latency* *T1 ± SE*	*Duration* *T2 ± SE*	*Sacc. End* *(T1+T2) ± SE*	*Amplitude* *L ± SE*	*Amplitude* *R ± SE*	*ISL*’ *T1+T2+T3*	*ISL*	*ISL’ -ISL*	*T3* *ISL’-(T1+T2)*
S1	123±2	39 ± 0.1	160 ± 2	7.47 ± 0.2	7.57 ± 0.1	205	196.7	8.3	43
S2	120.3 ± 2.3	38 ± 0.1	158 ± 2.2	7.5 ± 0.5	7.2 ± 0.1	196	183.1	12.9	38
S3	138 ± 4.2	42 ± 0.5	180 ± 3.9	8 ± 0.1	7.1 ± 0.1	218	207.1	10.9	38
S4	127.4± 1.3	37.6± 0.2	165± 1.4	8.4 ± 0.15	7.4 ± 0.2	218	223.3	5.3	53
S5	117 ± 2.3	38 ± 0.2	155 ± 2	7 ± 0.2	6.7 ± 0.2	189	198.7	-9.7	34
S6	107±1.5	43.5 ± 0.3	150.5 ± 1.4	7.6 ± 0.25	7.8 ± 0.24	187	192.7	-5.7	37

The times T1 (i.e., the time required to detect the stimulus and prepare the saccadic eye movement), T2 (i.e., the saccade duration) and T3 (i.e., the remaining stimulus time needed to process the visual stimulus) are defined in Fig 1. Values for T1, T2, T1+T2 and saccade amplitudes are given as means ± SEs based on three repeats of the same test (note that saccade amplitudes are listed separately for stimulus locations to the Right (R) and the Left (L) side of fixation). The ISL’ thresholds that correspond to 71% probability of a correct response were computed for each subject from the corresponding probability of correct response curve, whereas the ISL values are the results generated by the EMAIL test.

**Table 2:** Summary of results measured with a stimulus of 15% contrast at 0° and 8° eccentricity.

*Contrast 15%*	*Latency* *T1 ± SE*	*Duration* *T2 ± SE*	*Sacc. End* *(T1+T2) ± SE*	*Amplitude* *L ± std*	*Amplitude* *R ± std*	*ISL’ at 8°* *T1+T2+T3*	*T3* *ISL’- (T1+T2)*	*ISL_15_’ at* *0°*
S1	176.4 ± 5.5	38 ± 0.36	214±5.7	7.8 ± 0.14	7.2 ± 0.14	315.0	101	45
S2	155.4 ± 2.2	36.7 ± 0.1	192 ± 2.2	7.4 ± 0.3	6.9 ± 0.1	297	105.0	40
S3	185.1 ± 2.3	42.5 ± 0.5	227.5 ± 2	8.3 ± 0.2	7.1 ± 0.2	349	121.5	46.5
S4	172± 4.5	38.4± 0.1	210 ± 4.7	7.9 ± 0.2	6.65 ± 0.4	376	166	46
S5	148 ± 0.5	39.1 ± 0.7	187.1 ± 1.2	7.9 ± 0.5	7 ± 0.1	300	113	35
S6	161.3 ± 2.7	43.2 ± 0.3	204.4±2.7	7.9 ± 0.1	7.7 ± 0.2	315.0	111.0	57

All times are shown in ms. Values for T1, T2, T1+T2 and saccade amplitude are means ± SEs calculated from three repeat measurements and ISL´ threshold durations that correspond to 71% probability of a correct response as shown on each subject’s psychometric curve.

The question of interest is to establish whether T3 represents the time needed to process visual information in order to carry out the task successfully when saccades are involved, or whether full eyeball stability is required before the effective processing of retinal signals can take place. If the latter is the case, T3 overestimates the time needed to detect, localise, and register the position of the gap. In general, it is anticipated that saccadic suppression extends beyond the duration of the saccade. The occurrence of a brief lapse in post-saccadic visual processing has been attributed to the underdamping of interocular structures of the eye, such as the lens, as measured by the Purkinje-meter [4,5]. It is crucial to note, however, that the discrepancies in PSO durations arise from differing measurement techniques (i.e., lens versus pupil). As our study employs the EyeLink 1000, measuring PSOs based on pupil dynamics, our findings align well with previous studies that have employed similar methods and estimated PSO durations for similar saccade amplitudes, lasting between 25 and 35 ms [8,10]. In the analysis section, we observed a general pattern that emerged from the subject’s return velocities: across all subjects, when the eye position is at maximum (i.e., end of saccade), the damped oscillatory motion of the eyeball generates large angular speeds which exceed typical vergence drift velocities of < 20°/sec [19]. The peak return velocity was again subject dependent and ranged between 45°-160°/sec and lasted 17-33ms. Additionally, we found that abducting saccades across all subjects produced larger amplitudes (column 4:5, Tables 1 and 2) and in some subjects (four out of six), abducting saccades were found to produce significantly higher return velocities (e.g., 150 vs. 100°/sec) when compared to adducting saccades as shown in Fig 5 (right panel). This observation has been described before and the saccades of the abducting eye are known to produce larger PSOs [8,11,12,19]. This suggests that during PSOs there must be some asynchrony of action between the two eyes. Each eye undergoes high rotational speeds during the deceleration phase, and this can also affect position accuracy at the end of the saccade. Although visual sensitivity is maintained at high velocities, the ability to resolve fine spatial details is greatly diminished [23]. These observations may explain, at least in part, why the measured T3 durations for 15% contrast (Table 2) are significantly higher when compared to the T3 durations measured with 75% contrast stimuli (see Table 1). It is important to note that for all subjects, irrespective of contrast level, T3 (indicated by a coloured circle (75%) or a diamond (15%) in Fig 5, right panel) occurred after the eye approached its mean position (i.e., the eye velocity remained close to zero). These observations alone suggest that PSOs can have perceptual consequences since T3 extends beyond the time when the eye reaches its steady, mean position.

To confirm that this is the case, we measured the probability of a correct response for the 15% stimulus contrast presented at the point of regard (0°) to estimate directly the time needed to process the same visual attribute in the absence of saccadic eye movements (see methods for details). If PSOs do not delay processing of visual information, it would be reasonable to expect that the subject specific, T3 durations measured in the periphery should equal the time needed to process visual information in the absence of eye movements.

The use of the external timer to measure accurately the stimulus presentation time on the display reveals a systematic error of approximately one frame between the set time and the measured time in the majority of presentations. All measured ISL values were adjusted to account for this systematic error. EMAIL test results based on 12 repeats carried out on the same subject were used to estimate ISL mean and standard deviation values of 223.34 ± 6.7ms, respectively. Repeated estimates of ISL’ values measured using the external stimulus timer with fixed stimulus presentation times were also carried out with the same subject. The percentage correct response curves (similar to those shown in Fig 5) were used to estimate the ISL’ values that correspond to 71% correct response. This approach yielded ISL’ mean and standard deviation values of 225.17 ± 1.62ms. The difference between the two independent estimates, ISL’-ISL, is expected to exhibit a standard deviation of 6.9ms, i.e. (6.7^2^+1.62^2^)^0.5^. The differences between ISL’-ISL values shown above for six subjects fall well within the expected error range. The results show that the EMAIL test can be used on its own to measure ISLs and reveal the variability in saccade latencies, durations and visual processing times.

### Results for 0° eccentricity

**Fig 6:**
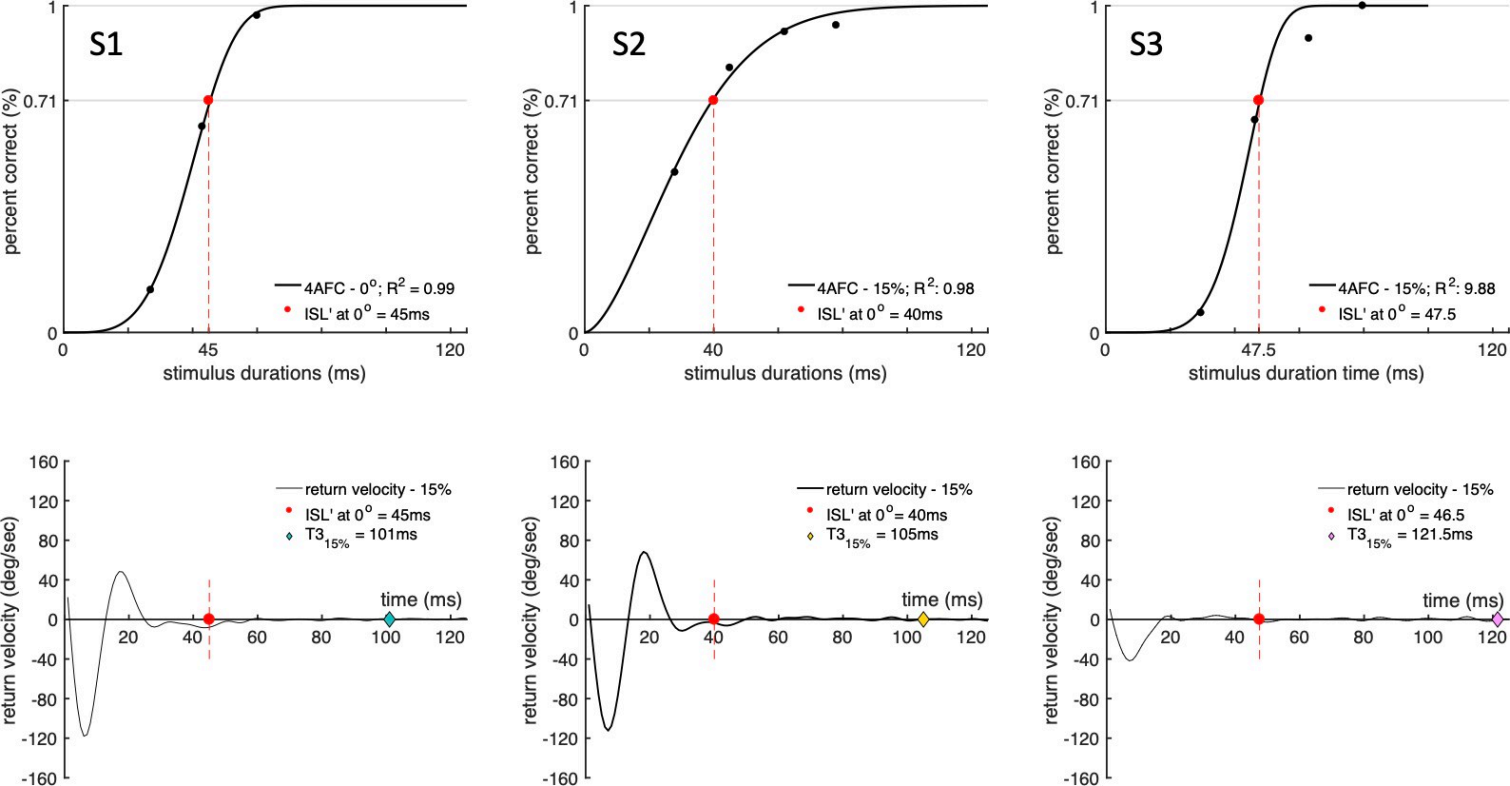
Examples of psychometric functions corrected for chance probability measured at the point of regard, under steady fixation, with stimuli of 15% contrast. Percentage correct responses and the corresponding return velocities are shown for three subjects. The stimulus presentation time, ISI’, each subject needs to achieve 71% correct response (in the absence of eye movements) is indicated in the top row by a red dot in each figure. For comparison, the same stimulus presentation times measured foveally at the point of regard are also plotted as red dots on the mean return velocity traces (in each of the lower figures) when the same stimulus was presented 8° in the periphery. In addition, the lower three figures also plot the measured ISL’ for 15% contrast stimuli at 8° eccentricity (coloured diamond). The results demonstrate that the time needed to process visual information at the end of each saccade is significantly longer when eye movements are involved.

The measurements with a stimulus contrast of 15% for the same visual stimulus presented centrally (0°) are listed in Table 2. We found that these thresholds are subject specific and ranged between 35-56ms (Fig 6, top row). In Fig 6 we show the measured psychometric functions at 0° for the same subjects for a stimulus contrast of 15%. For clarity of explanation, we also present each subject’s rightward return velocity templates derived from the measurements taken with the same stimulus presented peripherally at 8° eccentricity. We used 15% contrast simply because, as noted in experimental procedure, it was technically not possible for us to measure ISL’ at the point of regard with larger stimulus contrasts, due to the timing limitation imposed by the hardware.

The results shown in Fig 6 reveal the large increase in visual processing times measured 8° in the periphery (when eye movements are involved) when compared to those measured at the point of regard (under steady fixation). The observed increase is always greater than or equal to the equivalent PSO time. The time required to process the visual information of interest in the stimulus is longer when an eye movement is involved (see last two columns in Table 2). This finding suggests that eyeball instabilities at the end of saccades lengthen significantly the time needed to process the stimulus attribute of interest.

## Discussion and conclusions

We examined the time needed to process and encode the stimulus attribute of interest, both under steady fixation conditions and when saccadic eye movements are involved. The results show that the fixation durations needed to process stimulus-specific attributes at the end of a saccade are much longer than what is needed to carry out the same visual task under steady fixation conditions.

This discovery was made possible by measuring the “ISL” time the subject needs to process visual information at the end of saccades. The EMAIL test was designed primarily to investigate how mental health disorders affect oculomotor responses, including the time needed for the central processing of stimulus-specific attributes such as spatial structure, colour, motion and rapid flicker. In this study, we investigated how long the subject needs to view a peripheral stimulus at the end of a saccadic eye movement using a visual task that can only be carried out under foveal examination. We made use of visual crowding (see stimulus and distractors in Fig 1A) to ensure that the subject was unable to localise the position of the gap in a Landolt ring stimulus when presented at an eccentricity of 8° on either side of fixation. ‘Visual crowding’ describes the increase in contrast thresholds and the worsening of visual acuity when peripheral stimuli are surrounded by other similar stimuli [26,28]. The test does not require any eye-tracking equipment, but it allows for the inclusion of an eye-tracker to make it possible to delineate the contribution each component makes to the overall ISL time.

The immediate research question of interest was to establish whether eye movements affect cortical visual processing times in normal subjects. To answer this question, we investigated how eye movements and in particular the PSOs at the end of each saccade affect visual processing times.

The results show that both PSOs and the time needed to process and register the information present in a visual stimulus in order to be able to carry out the visual task are subject specific. When taken across all subjects, the T3 duration was found to always outlast the end of PSOs. We found that the shortest visual processing times needed to achieve 71% correct response were always accomplished after the eye had reached its mean position (Fig 5). In other words, the T3 times always extend beyond the end point of PSOs in all subjects investigated.

There are two possible explanations that may account for these findings. Saccadic suppression times that extend beyond the end point of a saccade would result in longer visual processing times. An alternative explanation would be that even in the absence of extended saccadic suppression times, the relative loss of spatial resolution due to image smear caused by eyeball instability during the PSO duration at the end of each saccade may also account for, or at least contribute to the apparent increase in visual processing time. The experimental findings also show that stimulus contrast affects both saccade latencies (T1) and visual processing times (ISL- (T1 +T2)). Equally important, the results show that PSO times remain relatively unaffected by stimulus contrast.

It is of interest to establish whether any pre-processing of visual information regarding the stimulus attribute of interest takes place during the time delineated by the end point of the saccade and the end point of PSOs. This kind of pre-processing would shorten the cortical processing time, when measured with respect to the PSO end point. To investigate this hypothesis, we carried out measurements of cortical processing times under conditions of steady fixation and compared the results with the equivalent times measured with respect to the PSO endpoint at the end of each saccade. This comparison could only be made for visual stimuli of low luminance contrast because of the limits on stimulus presentation time imposed by the equipment employed. The cortical processing times measured with respect to PSO end point (see Fig 5) were either similar or longer for all subjects when compared with equivalent results in the absence of eye movements. This observation is consistent with negligible or simply absent processing of the stimulus attribute during PSOs.

The second hypothesis involves image smear and the loss of spatial resolution during the PSO phase at the end of each saccade. Since the subject’s task in this investigation requires adequate spatial resolution to localise and register the gap in the Landolt ring, it is reasonable to assume that the processing of spatial information during PSOs is rendered ineffective by retinal image smear. It is well established that rapid movements of the retinal image cause loss of spatial resolution and that the effect is greater for stimuli of low luminance contrast [37–40]. Although the four minutes of arc Landolt ring gap size employed in this study is about four times larger than the average normal acuity limit [41], image smear can still cause a loss of spatial resolution, particularly when stimuli of low luminance contrast are employed. To test this hypothesis, we carried out a set of experiments to investigate how stimulus contrast affects cortical processing times. These experiments provided important results which show that the fast, damped, oscillatory phase which accompanies each saccade causes brief lapse in our ability to process visual information and that the delay involved is dependent on stimulus contrast (Fig 5) and may also not be stimulus specific [30]. The results are therefore consistent with the assumption that the human visual system cannot resolve fine spatial details when retinal images move at typical PSO speeds. We found that, in some subjects, the damped oscillatory phase can reach peak velocities of up to 160deg/sec (Fig 5). Indeed, at such high rotational speeds even targets defined by low spatial frequencies may be difficult to resolve [23]. This may well justify findings which suggest that suppression of retinal signals can outlast the end point of saccades [2,3,42]. Either neural suppression or significant loss of spatial resolution during PSOs will increase visual processing times. An extension of neural suppression to cover the oscillations of the eyeball at the end of each saccade may also have additional advantages by blocking out the confusing information contained in blurred retinal images during PSO times. The results suggest that both effects can contribute to the increased T3 durations measured in the current study.

It is also possible that errors in saccade landing positions can contribute to the longer T3 durations measured in this study. The stimulus employed is a cluster of rings and in such cases, goal directed saccades tend to end up at the ‘centre of gravity’ of the light flux distribution [18]. In this case, the end point of the saccade should correspond to the centre ring with the gap. In this study, the employed Landolt ring gap size is 4’ (i.e., four times larger than the average visual acuity [41]) and perfect foveation is not strictly needed to resolve the gap. There is, however, another factor that may have contributed to the large increase in visual processing times. Previous studies have shown that horizontal saccades do not exhibit perfect conjugacy, and with increasing eccentricity (>5°), the asymmetry between the two eyes also becomes more evident [11,12,43]. The lack of perfect conjugacy can affect binocular fixation by increasing transiently the image disparity between the two eyes. In this set of measurements, the observed variations in saccade amplitude were found to be small—ranging between 0.1° - 0.5°. However, when considering the task is visually demanding, requiring not only that the subject appropriately generates an eye movement, but also process the specific stimulus attribute at the end of the saccade, even small variations in landing positions can contribute to increased visual processing times, particularly when low contrast stimuli are employed. This could explain why T3 durations measured for 15% stimulus contrast at 8° eccentricity are found to be considerably longer than what one needs to carry out the same visual task when no eye movements are involved. This observation suggests that in addition to subject-specific, post-oscillatory movements, the fixation errors can also contribute to increased visual processing times. These findings are functionally important, given the fact that our eyes are always in motion. Also, when the subject’s task requires the processing of other stimulus attributes such as colour, rapid flicker or motion signals, the time needed to carry out visual tasks may vary significantly from those reported here, even when no eye-movements are involved [32].

In summary, this investigation shows how post-saccadic visual processing times are affected by the subject’s PSO durations, landing positions and stimulus contrast level. The measurement of eye-movements during the EMAIL test makes it possible to separate the three components that make up the ISL’ time. The EMAIL test uses only the staircase method without any eye-tracking equipment and measures the stimulus presentation time (ISL) the subject needs to achieve 71% correct response. The ISL’ time is computed from the probability of a correct response curve measured with fixed stimulus durations (selected to fall both above and below the ISL time). ISL’ represents the time needed to achieve the same probability of a correct response but takes much longer to carry out and the stimulus timing involves the use of additional hardware and expensive eye-tracking equipment which requires subject-specific calibration. The very good agreement between the two methods justifies the EMAIL test as an equivalent technique that can be employed to measure the combined saccadic response latencies and visual processing times. The results that emerged from this study are of practical significance and can be used to improve the design of cockpit displays and in other visually demanding occupations when specific information needs to be processed quickly and accurately [44]. When searching for specific targets, the use of a large display with stimuli of low luminance contrast generates longer visual search times [37,45]. The findings from this study suggest that smaller, task-specific displays and the use of high contrast stimuli can have significant advantages by minimizing the number of fixations needed to locate the target of interest [45] and by shortening significantly the ISL time associated with each saccade.

## Supporting information

S1 File (EDF)

S2 File (EDF)

S3 File (EDF)

S4 File (EDF)

S5 File (EDF)

S6 File (EDF)

S7 File (EDF)

S8 File (EDF)

S9 File (EDF)

S10 File (MAT)

S11 File (MAT)

S12 File (MAT)

S13 File (MAT)

S14 File (MAT)

S15 File (MAT)

S16 File (MAT)

S17 File (MAT)

S18 File (MAT)

S19 File (XML)

S20 File (XML)

S21 File (XML)

S22 File (XML)

S23 File (XML)

S24 File (XML)

S25 File (XML)

S26 File (XML)

S27 File (XML)
